# Digital capital and social alienation among undergraduate students in the United Arab Emirates

**DOI:** 10.3389/fpsyg.2026.1864357

**Published:** 2026-09-09

**Authors:** Rula Odeh Alsawalqa, Maitha Alawani, Amenh Almarzooqi, Shamma Aldhuhoori, Fatima Abdalla, Roqaya Alreesi

**Affiliations:** 1 Department of Sociology, University of Khorfakkan, Khorfakkan, United Arab Emirates; 2 Department of Sociology, The University of Jordan, Amman, Jordan

**Keywords:** digital capital, digital competencies, higher education, social alienation, United Arab Emirates

## Abstract

This study examined the relationship between digital capital and social alienation, conceptualized as a multidimensional construct, among undergraduate students in the United Arab Emirates. A cross-sectional survey design was employed using a convenience sample of 320 undergraduate students enrolled at a government-affiliated university in the Emirate of Sharjah, United Arab Emirates. Data were collected during the 2025–2026 academic year through a self-administered online questionnaire. The findings indicated high levels of digital capital and low levels of social alienation. At the dimensional level, meaninglessness emerged as the most prominent dimension of social alienation, followed by normlessness, powerlessness, and social isolation. Digital capital was significantly and negatively associated with overall social alienation and all of its dimensions, with the strongest association observed for normlessness, followed by social isolation, meaninglessness, and powerlessness. Multiple regression analysis further showed that digital capital remained significantly associated with social alienation after controlling for sex, age, and academic level (*β* = −0.61, *p* < 0.001), explaining 39.7% of the variance in social alienation. Significant differences in digital capital were found according to sex, favoring females, and academic level, with higher levels observed among senior students. No significant differences in digital capital were found across age groups, and no statistically significant differences in social alienation were observed across sex, age, or academic level. These findings suggest that digital capital may represent an important resource associated with lower levels of social alienation among university students within digitally mediated higher education environments. The findings further support the view that the potential contribution of digital capital extends beyond the acquisition of digital competencies and may be associated with broader processes of academic and social integration.

## Introduction

In digitally mediated academic environments, university students’ experiences of connection, belonging, and engagement are increasingly shaped by their ability to effectively navigate digital technologies. Although these environments provide expanded opportunities for communication and participation, they may also be associated with adverse psychological experiences, including loneliness and social disconnection (Smith and Alheneidi, 2023; Ko et al., 2022). Such experiences reflect broader processes of perceived disconnection and are closely related to social alienation, which encompasses disruptions in individuals’ relationships with their social environment and includes dimensions such as social isolation, meaninglessness, and powerlessness (Seeman, 1959). This perspective is consistent with research emphasizing the fundamental human need for belonging as a core component of psychological well-being and social functioning (Baumeister and Leary, 1995). Accordingly, understanding the factors associated with students’ experiences of social connectedness and alienation has become an important concern within contemporary personality and social psychology.

### Digital capital in digitally mediated higher education

Within this context, digital capital has emerged as a contemporary extension of classical forms of capital that shape individuals’ opportunities and life chances in the digital age. Rooted in Bourdieu’s theory of capital, which conceptualizes economic, cultural, and social capital as accumulable and transferable resources that individuals mobilize to maintain or enhance their social position (Bourdieu, 1986; Alrawashdeh, 2025), digital capital has been defined as the accumulation of digital competencies and digital technologies, whereby digital competencies represent internalized abilities and aptitudes and digital technologies constitute externalized resources (Ragnedda, 2018). As a bridge between online and offline life chances, digital capital enables individuals to convert digital competencies and technologies into other forms of capital, thereby enhancing their opportunities to benefit from digital engagement (Ragnedda, 2018; Ragnedda et al., 2020). While this theoretical conceptualization encompasses both digital competencies and digital technologies, the present study adopts a competence-based operationalization of digital capital. Specifically, digital capital is assessed through students’ digital competencies across five domains: information and data literacy, communication and collaboration, digital content creation, safety, and problem solving (Addeo et al., 2023). Therefore, the findings should be interpreted within this competence-based operationalization rather than as representing the broader theoretical construct of digital capital.

Building upon this conceptualization, a Bourdieusian perspective further emphasizes that digital capital is conceptualized as the accumulation of digital competencies and digital technologies that can be converted into other forms of capital, thereby enhancing individuals’ opportunities to benefit from digital engagement (Ragnedda, 2018; Ragnedda and Ruiu, 2020). Rather than functioning in isolation, digital capital connects individuals’ existing economic, cultural, and social capital with opportunities available in digital environments, facilitating the conversion of digital capital into broader social, cultural, and economic advantages (Ragnedda, 2018; Lybeck et al., 2023). Recent theoretical developments further conceptualize digital capital as a relational and field-dependent form of capital whose value depends on how digital competencies and technologies are accumulated, recognized, and converted within specific social contexts. In this process, digital capital interacts with habitus and other forms of capital to shape individuals’ capacity to benefit from digital opportunities and influence their social trajectories (Ragnedda et al., 2025). Consequently, digital capital provides a valuable framework for explaining how social inequalities may be reproduced, transformed, or, under certain conditions, reduced within digitally mediated societies (Lybeck et al., 2023).

Importantly, contemporary scholarship emphasizes that the value of digital technologies lies not in access alone but in individuals’ capacity to develop digital competencies and convert digital opportunities into meaningful social, educational, and psychological outcomes (Ragnedda, 2018; Ragnedda et al., 2020). Accordingly, individuals with similar levels of digital access may differ substantially in their ability to mobilize digital capital and derive benefits from digital engagement (van Deursen and van Dijk, 2019; Rodríguez-Camacho et al., 2024). In this sense, digital capital is understood as a dynamic construct comprising digital competencies and resources that are accumulated over time and continuously shaped by learning, experience, and broader structural conditions, including access to infrastructure, institutional support, and opportunities for meaningful digital participation (Ragnedda and Ruiu, 2020; Park, 2017).

From a psychological perspective, digital capital may foster students’ sense of competence and social connectedness, two fundamental psychological needs identified in Self-Determination Theory (Deci and Ryan, 2000). These perspectives are presented solely to provide a conceptual framework for interpreting the potential relationship between digital capital and social alienation and do not represent mechanisms directly examined in the present study.

This perspective is particularly relevant in the United Arab Emirates, where advanced digital infrastructure and near-universal internet connectivity have largely minimized disparities in basic digital access, with internet users representing approximately 99% of the population (Telecommunications and Digital Government Regulatory Authority, 2023). In such a digitally mature context, differences among university students are more likely to reflect variations in digital competencies and the ability to effectively mobilize digital resources than disparities in technology access (van Deursen and Helsper, 2015; Rodríguez-Camacho et al., 2024). Consistent with this view, recent national evidence from the UAE showed that while digital access did not differ significantly across educational levels, digital competence varied according to educational attainment, age, field of work, and Emirate of residence (Muschert and Shomotova, 2025). These findings reinforce the importance of examining digital capital beyond access alone and provide a strong rationale for investigating its relationship with social capital and social alienation among university students in the UAE.

Within higher education, students’ academic and social lives are increasingly embedded in digitally mediated environments. Consequently, the ability to mobilize digital capital has been associated with academic engagement, social integration, and psychological well-being (Ragnedda and Ruiu, 2020; Rodríguez-Camacho et al., 2024). Effective engagement in digital environments has also been linked to stronger social connectedness and perceived social support, factors conceptually related to lower levels of social alienation (Baumeister and Leary, 1995; Diener et al., 2018).

### Social alienation in digitally mediated university environments

While digital technologies expand opportunities for learning and interaction, they also introduce new forms of inequality and may be associated with diminished quality of social interactions and increased feelings of loneliness and social disconnection, particularly when patterns of digital engagement are passive or fragmented (Kross et al., 2013; Verduyn et al., 2017). These patterns suggest that digital environments can simultaneously facilitate and hinder meaningful social experiences, positioning digital capital as a potentially important factor associated with students’ sense of social connectedness, belonging, and vulnerability to experiences of social alienation within digitally mediated contexts.

In parallel, social alienation is increasingly conceptualized as a multidimensional psychological experience reflecting a perceived disconnection between individuals and their social environment (Perveen et al., 2021; Liu et al., 2025). Although the concept originates in classical sociology, contemporary perspectives emphasize its subjective and experiential nature, particularly in relation to individuals’ perceived sense of control, meaning, and social belonging (Seeman, 1959; Baumeister and Leary, 1995). In this context, Seeman (1959) operationalized alienation into four key dimensions: powerlessness, meaninglessness, normlessness, and social isolation. These dimensions represent four interrelated aspects of perceived disconnection, including diminished personal agency, difficulties in constructing meaning, uncertainty regarding social expectations, and a weakened sense of connectedness with others. Empirical research has consistently linked these experiences to adverse psychological outcomes, including increased distress, loneliness, and reduced well-being (Perveen et al., 2021; Chang et al., 2025; Verduyn et al., 2017).

Seeman’s four dimensions provide a useful framework for understanding how social alienation may manifest within digitally mediated university environments. Students may experience powerlessness when navigating complex digital systems that require advanced competencies, meaninglessness when exposed to fragmented and overwhelming information, normlessness in online environments characterized by rapidly evolving social norms, and social isolation when digitally mediated interactions fail to satisfy their need for belonging despite frequent online connectivity (Seeman, 1959; Turkle, 2011; Verduyn et al., 2017). In digitally mediated environments, these dimensions may manifest in distinct and psychologically meaningful ways. Students may experience powerlessness when navigating complex digital systems and institutional platforms that require advanced competencies. Meaninglessness may emerge from exposure to fragmented and overwhelming information environments that hinder coherent understanding. Normlessness may arise in online contexts characterized by fluid and rapidly shifting norms and reduced social cues, whereas social isolation may persist despite high levels of connectivity, particularly when digital interactions remain superficial and fail to satisfy individuals’ fundamental need for belonging (Turkle, 2011; Verduyn et al., 2017).

These dynamics are particularly salient in university settings, where students navigate complex academic and social demands within digitally intensive environments. Empirical evidence indicates that experiences of social alienation among university students are associated with lower academic engagement, reduced life satisfaction, and increased psychological distress (Alkouri et al., 2022; Perveen et al., 2021; Chang et al., 2025).

Moreover, patterns of digital engagement have been associated with students’ experiences of social alienation. Unbalanced or excessive use of digital media has been linked to increased loneliness and social withdrawal, whereas digital exclusion or limited opportunities for meaningful digital participation may exacerbate feelings of psychological isolation (Smith and Alheneidi, 2023; Zhong and Wang, 2025; Tabassum et al., 2024). These findings reinforce the importance of examining digital capital in relation to students’ social alienation within digitally mediated university environments.

### Digital capital and social alienation: theoretical and empirical linkages

Empirical evidence from Arab academic contexts further highlights the broader psychological and social implications of digital capital. For instance, findings reported by Alrawashdeh (2025), conducted among undergraduate students within an Arab university setting, indicated that higher levels of digital capital were associated with lower exposure to cyberbullying and higher levels of perceived digital social support. These findings suggest that digital capital may support students’ ability to develop supportive online networks, strengthen their sense of connectedness, and navigate digital environments more effectively.

Although these findings originate from the context of cyberbullying, they point more broadly to the potential relevance of digital capital as a resource associated with lower levels of adverse social and psychological experiences in digitally mediated environments. By supporting social connectedness, digital capital may be associated with lower vulnerability to feelings of isolation and disconnection, which constitute central components of social alienation.

Accordingly, the relationship between digital capital and social alienation is of both theoretical and practical importance. The theoretical perspectives discussed above are used to contextualize and interpret the observed associations rather than to propose or test specific mediating mechanisms within the present study. Within this conceptual framework, higher levels of digital capital may function as a resource associated with greater perceived self-efficacy, more adaptive patterns of engagement, and stronger feelings of social connectedness and belonging (Bandura, 1997; Baumeister and Leary, 1995). Emerging evidence suggests that experiences within digitally mediated social environments may be associated with psychological well-being and identity-related processes (Valkenburg et al., 2022; Yang et al., 2018). In particular, patterns of social media use and online social comparison may have implications for individuals’ well-being and identity clarity, although these associations vary according to the nature of digital experiences and individual characteristics.

Conversely, limited digital competencies or unequal access to digital capital “digital resources” may undermine individuals’ sense of control, restrict opportunities for identity expression, and hinder effective participation in digitally mediated environments. Such constraints may be associated with increased vulnerability to social exclusion, loneliness, and feelings of isolation (Ko et al., 2022; Smith and Alheneidi, 2023) and may correspond to experiences that reflect core dimensions of social alienation, including powerlessness, meaninglessness, normlessness, and social isolation.

Taken together, the preceding theoretical perspectives and empirical findings suggest that digital capital extends beyond the acquisition of digital competencies and may constitute an important resource associated with lower levels of social alienation among university students. Drawing on Bourdieu’s theory of capital, the digital divide framework, and prior empirical evidence, the available literature suggests that higher levels of digital capital are likely to be associated with lower levels of social alienation and its dimensions among university students. More specifically, students with higher levels of digital capital, reflected in stronger digital competencies, may be expected to report lower levels of powerlessness, meaninglessness, normlessness, and social isolation. These theoretically and empirically informed expectations guided the development of the research questions and subsequent empirical analyses.

Previous research has suggested that demographic characteristics, particularly sex, age, and academic level, may be associated with differences in digital capital (Ragnedda et al., 2020; Rodríguez-Camacho et al., 2024) as well as students’ experiences of social alienation within higher education (Altaj and Al-Mawajdeh, 2020; Belikova and Pronenko, 2023). Accordingly, these demographic variables were included as control variables to examine whether the association between digital capital and social alienation remained significant after accounting for key demographic differences among university students.

Collectively, these theoretical perspectives provide a coherent conceptual framework for the present study. Bourdieu’s theory explains digital capital as a resource that can be accumulated and converted into social advantages, the digital capital framework specifies the competencies through which these resources are mobilized in digitally mediated environments, and Seeman’s conceptualization of alienation provides the psychosocial framework for understanding how differences in digital capital may be associated with experiences of powerlessness, meaninglessness, normlessness, and social isolation. Together, these perspectives offer a complementary explanation of why digital capital may be associated with lower levels of social alienation among university students.

### Research gap and study objectives

Despite growing scholarly interest in digital inequalities and students’ psychosocial experiences, empirical research on digital capital remains fragmented, particularly regarding its psychosocial implications among university students. Existing studies have primarily examined digital capital in relation to digital participation, educational engagement, digital competencies, social support, sense of belonging, and broader forms of digital inequality (Ragnedda, 2018; Clamor and Saloma, 2023; Rodríguez-Camacho et al., 2024; Alrawashdeh, 2025; Muschert and Shomotova, 2025). In parallel, research on social alienation has largely focused on its associations with psychological distress, loneliness, life satisfaction, academic engagement, and social adjustment (Perveen et al., 2021; Chang et al., 2025; Liu et al., 2025). Although these two bodies of literature collectively suggest that digital capital is closely associated with individuals’ psychosocial experiences, they have evolved largely in parallel and have rarely been integrated within a unified explanatory framework.

Moreover, recent scholarship has begun to examine forms of alienation emerging within digital environments, including technology-related alienation and digital labor alienation (Pan et al., 2023). However, these investigations have primarily focused on economic exploitation and digitally mediated labor processes rather than on multidimensional social alienation encompassing powerlessness, meaninglessness, normlessness, and social isolation (Seeman, 1959). Consequently, little is known about whether digital capital is associated with multidimensional social alienation among university students.

This gap is particularly evident in Arab higher education contexts and is especially pronounced in the United Arab Emirates. Existing research in the UAE has primarily examined digital capital and its demographic and socioeconomic correlates (Muschert and Shomotova, 2025). Although this work provides important evidence that digital capital is embedded within broader patterns of social stratification in the UAE, it does not examine whether differences in digital capital are associated with students’ psychosocial experiences, particularly multidimensional social alienation. Furthermore, prior studies have generally examined digital capital in relation to discrete psychosocial outcomes, such as social support, sense of belonging, cyberbullying, and well-being, rather than investigating its relationship with multidimensional social alienation within a unified conceptual framework (Clamor and Saloma, 2023; Alrawashdeh, 2025; Rodríguez-Camacho et al., 2024). Accordingly, the relationship between digital capital and multidimensional social alienation remains insufficiently understood.

The principal contribution of the present study lies in integrating digital capital and multidimensional social alienation within a single empirical framework in higher education. By examining competence-based digital capital in relation to powerlessness, meaninglessness, normlessness, and social isolation, the study extends existing research beyond digital access, participation, and discrete psychosocial outcomes to consider students’ broader experiences of meaning, normative clarity, and social connectedness. It also provides empirical evidence from an underrepresented Arab higher education context characterized by advanced digital infrastructure and near-universal internet penetration (Telecommunications and Digital Government Regulatory Authority, 2023), where inequalities may be reflected less in basic access than in students’ capacities to mobilize digital competencies effectively. Accordingly, the present study investigates the relationship between digital capital and multidimensional social alienation among undergraduate students at one university in the Emirate of Sharjah, United Arab Emirates.

### Research question

1. What is the association between digital capital and social alienation among university students?
2. What are the levels of digital capital and social alienation, including its dimensions (powerlessness, meaninglessness, normlessness, and social isolation), among university students?
3. Are there statistically significant differences in digital capital and social alienation based on demographic and academic characteristics (sex, age, and academic level)?
4. To what extent is digital capital associated with social alienation among university students when controlling for demographic and academic characteristics (sex, age, and academic level)?
5. What is the strength and direction of the associations between digital capital and each dimension of social alienation?

## Methods

### Participant recruitment and data collection

This study employed a cross-sectional survey design conducted at a government-affiliated university in the Emirate of Sharjah, United Arab Emirates. The study population consisted of all undergraduate students enrolled during the 2025–2026 academic year (*N* = 1,630), including 909 females and 721 males according to official university enrollment statistics. The student population was characterized by a degree of relative social and cultural homogeneity, as most students originated from local communities within the Emirate of Sharjah. This relative homogeneity may have contributed to similarities in patterns of technology use and social interaction and should therefore be considered when interpreting the findings.

A convenience sampling technique was employed to recruit participants due to contextual constraints, including the shift to remote learning, which limited access to comprehensive sampling frames and hindered the implementation of probability sampling methods. Convenience sampling was considered an appropriate methodological option under these circumstances because it enabled the practical and flexible collection of data while maintaining an acceptable level of representation, particularly in exploratory social research. To evaluate the adequacy of the sample size, the study was guided by the recommendations of Krejcie and Morgan (1970). For a study population of 1,630 students, the recommended minimum sample size was approximately 310 participants at a 95% confidence level and a 5% margin of error. Accordingly, the final sample of 320 participants was considered adequate for the planned statistical analyses.

The final sample consisted of 320 undergraduate students, including 144 males (45.0%) and 176 females (55.0%). In terms of academic level, the largest proportion of participants were in their fourth year (38.4%), followed by third-year students (30.3%), second-year students (18.4%), and first-year students (12.8%). Regarding age distribution, nearly half of the participants (48.1%) were aged between 21 and 25 years, while 24.0% were above 30 years, 15.4% were 20 years or younger, and 12.5% were between 26 and 30 years. These characteristics indicate a relatively heterogeneous sample in terms of academic standing and age distribution.

Data were collected during the second semester of the 2025–2026 academic year using an online questionnaire administered via Google Forms. The survey link was distributed to approximately 810 undergraduate students through their institutional accounts on the university’s learning management platforms and through WhatsApp groups associated with academic departments. A total of 320 completed questionnaires were received, yielding a response rate of 39.5%. The online survey required participants to sign in using their institutional university accounts, and the survey settings restricted submissions to one response per participant, thereby minimizing the possibility of duplicate responses. At the beginning of the questionnaire, participants were first asked whether they were willing to participate in the study. Those who selected “Yes” were directed to an introductory information page describing the study title, objectives, procedures, voluntary nature of participation, and confidentiality assurances before proceeding to the questionnaire. The online questionnaire was configured so that all survey items were mandatory. Consequently, all submitted questionnaires were complete, and the final dataset contained no missing or incomplete responses. Nevertheless, the use of an online, self-administered survey may have introduced self-selection bias due to the voluntary nature of participation.

Ethical approval for this study was obtained from the Research Committee of the Department of Sociology at the University of Khorfakkan, United Arab Emirates (Approval No. 2/2026). Participation was entirely voluntary. Because the study involved an anonymous online questionnaire and no personally identifying information was collected, written informed consent was not required. Informed consent was implied through participants’ voluntary completion of the questionnaire following review of the introductory information page.

### Measures

Digital Capital Scale: Digital capital was measured using an 18-item self-report instrument derived from the digital competence framework reported by Addeo et al. (2023). The Arabic version of the instrument was adopted from Alrawashdeh (2025), who previously administered the scale among university students in an Arab educational context. Because the present study was conducted in Arabic, the questionnaire was administered using the existing Arabic version. The instrument had previously undergone forward and back translation procedures conducted by specialists in English language and translation at a public university. To further ensure conceptual and linguistic equivalence, the research team reviewed the Arabic items against the original English version reported by Addeo et al. (2023) and verified the consistency of meanings across both versions. No items were added, omitted, or substantively modified.

The instrument comprises 18 items covering five domains of digital competence: information and data literacy, communication and collaboration, digital content creation, safety, and problem solving. Responses were recorded on a five-point Likert scale ranging from 1 (strongly disagree) to 5 (strongly agree). Although the instrument assesses five domains, the items were aggregated to generate a single composite score representing overall digital capital, consistent with the conceptualization of digital capital as an integrated and accumulative resource (Ragnedda, 2018; Ragnedda et al., 2020). Higher scores indicate higher levels of digital capital.

Conceptually, digital capital encompasses both digital technologies and digital competencies that can be mobilized to generate meaningful social, educational, and economic outcomes (Ragnedda, 2018; Ragnedda et al., 2020). However, for the purposes of the present study, digital capital was operationally defined as university students’ overall capacity to effectively mobilize digital competencies across information and data literacy, communication and collaboration, digital content creation, safety, and problem solving within digitally mediated environments. This operationalization was considered particularly appropriate for the United Arab Emirates context, which is characterized by advanced digital infrastructure and exceptionally high levels of internet connectivity. According to the Telecommunications and Digital Government Regulatory Authority, active internet users constitute approximately 99% of the UAE population, reflecting near-universal digital access (Telecommunications and Digital Government Regulatory Authority, 2023). In addition, national digital transformation initiatives and digital government enablers have expanded the integration of digital technologies across public, private, and educational sectors in the UAE (Telecommunications and Digital Government Regulatory Authority, 2023; UAE Government, 2025). In such a digitally mature environment, differences among university students are more likely to emerge in their ability to effectively utilize digital resources and transform digital opportunities into meaningful academic and social outcomes than in basic access to digital technologies.

Social Alienation Scale: Social alienation was measured using a self-report instrument grounded in the classical conceptualization of alienation proposed by Seeman (1959) and based primarily on Dean’s Alienation Scale (DAS; Dean, 1961). The original DAS consists of 24 items assessing three dimensions of alienation—powerlessness, normlessness, and social isolation—which operationalize three of Seeman’s five conceptual meanings of alienation.

Because the present study was conducted in Arabic, the questionnaire was administered in Arabic. The Arabic version of the DAS had previously undergone forward and back translation procedures conducted by specialists in English language and translation at a public university. To further ensure conceptual and linguistic equivalence, the research team reviewed the translated items against the original English version and compared them with existing Arabic adaptations of the scale. No items were added, omitted, or substantively modified.

To provide a more comprehensive assessment of social alienation, the present study additionally incorporated the meaninglessness dimension from the instrument developed by Rayce et al. (2017). Meaninglessness represents one of Seeman’s original conceptual meanings of alienation but is not operationalized as a distinct subscale in the original DAS. This dimension comprised eight items assessing individuals’ perceived lack of meaning and purpose in life. Accordingly, the final instrument consisted of 32 items distributed across four dimensions: powerlessness, normlessness, social isolation, and meaninglessness.

Responses were recorded on a five-point Likert scale ranging from 1 (strongly disagree) to 5 (strongly agree), with higher scores indicating higher levels of perceived social alienation. The use of the DAS in the present study was considered appropriate because it represents one of the most widely recognized multidimensional measures of alienation in sociological and psychological research. Moreover, the scale has previously been translated, culturally adapted, and administered among Arab university student populations, including Kuwaiti university students, supporting its applicability within Arab higher education contexts (Al-Tarah and Al-Kandari, 1992).

Prior to the main data collection, the questionnaire was reviewed by experts in sociology and social research from universities in Jordan and the United Arab Emirates to evaluate the clarity, relevance, and cultural appropriateness of the items. Subsequently, a pilot study involving 60 university students was conducted to assess item comprehensibility and preliminary reliability. The pilot findings indicated satisfactory internal consistency, supporting the suitability of the questionnaire for administration in the main study.

### Statistical analysis

Statistical analyses were conducted using IBM SPSS Statistics (Version 25). Descriptive statistics, including means and standard deviations, were computed to examine the levels of digital capital and social alienation and their respective dimensions. Data normality was assessed using skewness and kurtosis indices. All values fell within acceptable ranges (±1 for skewness and ±1.96 for kurtosis), supporting the use of parametric statistical analyses. In addition, the normality of residuals was examined and found to be satisfactory. To assess the potential influence of common method variance resulting from the use of self-report measures collected from the same participants at a single point in time, Harman’s single-factor test was conducted using an unrotated exploratory factor analysis.

To examine the relationships between digital capital and the dimensions of social alienation, Pearson’s correlation coefficients were calculated, with the level of statistical significance set at *α* = 0.05.

Differences in the study variables according to demographic and academic characteristics (sex, age, and academic level) were examined using independent-samples t-tests and one-way analyses of variance (ANOVA). The assumption of homogeneity of variance was evaluated using Levene’s test. When statistically significant differences were identified, Tukey’s *post hoc* test was performed to determine the direction of these differences. In addition to statistical significance, effect sizes were calculated using Cohen’s d for t-tests and eta squared (η^2^) for ANOVA.

To further examine the relationship between digital capital and social alienation while controlling for demographic variables (sex, age, and academic level), multiple regression analysis was performed. In the regression model, sex was coded as 0 for males and 1 for females. Age was represented by four ordered categories (≤20 years, 21–25 years, 26–30 years, and >30 years), whereas academic level reflected students’ progression from the first to the fourth year of study. The significance criterion and reporting of effect size estimates followed the procedures described above. Prior to conducting the regression analysis, the assumptions of linearity, normality of residuals, homoscedasticity, and multicollinearity were examined and found to be satisfactory. Multicollinearity was assessed using variance inflation factor (VIF) values, all of which were below the recommended threshold of 5, indicating no evidence of multicollinearity.

## Results

### Descriptive statistics of main study variables

Descriptive analyses indicated that students reported high levels of digital capital (M = 3.79, SD = 0.73) and low levels of social alienation (M = 2.30, SD = 0.64). At the dimensional level, all components of social alienation were low, with meaninglessness ranking highest (M = 2.41, SD = 0.65), followed by normlessness (M = 2.32, SD = 0.71), powerlessness (M = 2.21, SD = 0.62), and social isolation as the lowest (M = 2.18, SD = 0.59; Table 1).

**Table 1:** Descriptive statistics, internal consistency, and data screening indicators.

Variable	M	SD	Α	Item-total correlations	Skewness	Kurtosis
Digital capital	3.79	0.73	0.92	0.518–0.744	0.12	−0.45
Social alienation (Total)	2.30	0.64	0.93	0.450–0.822	−0.25	−0.25
Powerlessness	2.21	0.62	0.91	0.458–0.732	−0.18	0.42
Normlessness	2.32	0.71	0.91	0.637–0.711	0.10	−0.12
Social isolation	2.18	0.59	0.93	0.450–0.656	−0.05	0.55
Meaninglessness	2.41	0.65	0.92	0.728–0.822	0.08	−0.22

M, Mean; SD, Standard Deviation; α, Cronbach’s alpha. Item-total correlations represent the range of corrected item-total correlations for each scale. All skewness values were within ±1 and all kurtosis values were within ±1.96, supporting the assumption of normality.

### Psychometric properties and data screening

Table 1 presents descriptive statistics, reliability coefficients, item-total correlations, and normality indicators for all study variables. High internal consistency was observed across all measures, with Cronbach’s alpha coefficients of 0.92 for digital capital and 0.93 for social alienation. The sub-dimensions of social alienation also demonstrated high internal consistency, with Cronbach’s alpha coefficients ranging from 0.91 to 0.93. Evidence of item homogeneity was further supported by statistically significant item-total correlations. For digital capital, item-total correlations ranged from 0.518 to 0.744. For the dimensions of social alienation, item-total correlations ranged from 0.458 to 0.732 for powerlessness, 0.637 to 0.711 for normlessness, 0.450 to 0.656 for social isolation, and 0.728 to 0.822 for meaninglessness. All correlations exceeded the commonly recommended minimum threshold of 0.30, indicating satisfactory coherence between individual items and their respective dimensions. However, these correlations should not be interpreted as definitive evidence of construct validity or confirmation of the proposed dimensional structure.

As an additional assessment of common method variance, Harman’s single-factor test was conducted by entering all measurement items into an unrotated exploratory factor analysis. The first unrotated factor accounted for 36.7% of the total variance, which is below the commonly recommended threshold of 50%, suggesting that common method variance was unlikely to substantially influence the observed relationships. In addition, the assumption of normality was satisfied, as all skewness values fell within ±1 and all kurtosis values fell within ±1.96, supporting the use of parametric statistical analyses.

### Relationship between digital capital and social alienation

Pearson’s correlation analysis (Table 2) revealed statistically significant negative associations between digital capital and social alienation across all dimensions, with correlation coefficients ranging from moderate to strong. The strongest negative association was observed for normlessness (*r* = −0.69, *p* < 0.001), followed by social isolation (*r* = −0.68, *p* < 0.001) and meaninglessness (*r* = −0.66, *p* < 0.001), whereas a comparatively weaker, yet still substantial, association was found for powerlessness (*r* = −0.55, *p* < 0.001). Digital capital was also negatively associated with overall social alienation (*r* = −0.63, *p* < 0.001). Positive and moderate intercorrelations were observed among the dimensions of social alienation, suggesting that the dimensions were related but conceptually distinct.

**Table 2:** Pearson correlations between digital capital and dimensions of social alienation.

Variable	1	2	3	4	5	6
Digital capital	—					
Powerlessness	−0.55***	—				
Normlessness	−0.69***	0.62***	—			
Social isolation	−0.68***	0.59***	0.65***	—		
Meaninglessness	−0.66***	0.60***	0.67***	0.63***	—	
Social alienation	−0.63***	0.78***	0.81***	0.79***	0.80***	—

***p < 0.001.

### Group differences in digital capital and social alienation

Independent samples t-test results (Table 3) showed a statistically significant difference in digital capital between male and female students (t = −4.85, *p* < 0.001), with females reporting higher levels (M = 3.96) compared to males (M = 3.58). The effect size was moderate (Cohen’s d = 0.54). However, no statistically significant differences were found in social alienation based on sex (t = 1.47, *p* = 0.140). One-way ANOVA results indicated no statistically significant differences in digital capital across age groups (*F* = 1.156, *p* = 0.278), nor in social alienation (*F* = 1.497, *p* = 0.060), although the latter approached statistical significance.

**Table 3:** Group differences in digital capital and social alienation.

Variable	Group/test	Statistic (t/F)	P	Effect size	*Post-hoc* (Tukey)
Digital Capital	Sex (t)	t = −4.85	*p* < 0.001***	d = 0.54	—
Social Alienation	Sex (t)	t = 1.47	*p* = 0.140	d = 0.16	—
Digital Capital	Age (F)	*F* = 1.16	*p* = 0.278	η^2^ = 0.01	—
Social Alienation	Age (F)	*F* = 1.50	*p* = 0.060	η^2^ = 0.02	—
Digital Capital	Academic Level (F)	*F* = 10.81	*p* < 0.001***	η^2^ = 0.08	1 < 3**, 1 < 4***, 2 < 4**
Social Alienation	Academic Level (F)	*F* = 1.71	*p* = 0.165	η^2^ = 0.02	—

t, independent samples t-test; F, one-way ANOVA; d = Cohen’s d; η^2^, eta squared. Post hoc comparisons were conducted using Tukey’s test. Significant differences were found between first-year students and both third- and fourth-year students, as well as between second- and fourth-year students. *p < 0.05, **p < 0.01, ***p < 0.001.

Significant differences were found in digital capital across academic levels (*F* = 10.805, *p* < 0.001), with a moderate effect size (η^2^ = 0.08). *Post hoc* comparisons using Tukey’s test revealed that students in higher academic levels, particularly those in the third and fourth years, reported significantly higher levels of digital capital compared to lower-level students. No statistically significant differences were found in social alienation across academic levels (*F* = 1.710, *p* = 0.165).

### Regression analysis

A multiple regression analysis was conducted to examine the association between digital capital and social alienation while controlling for demographic variables (sex, age, and academic level). The overall model (Table 4) was statistically significant, *F*(4, 315) = 52.40, *p* < 0.001, accounting for approximately 39.7% of the variance in social alienation (R^2^ = 0.397, Adjusted R^2^ = 0.390), Digital capital demonstrated a strong and statistically significant negative association with social alienation (*β* = −0.61, B = −0.58, 95% CI [−0.68, −0.48], *p* < 0.001), indicating that higher levels of digital capital were associated with lower levels of perceived social alienation among students.

**Table 4:** Multiple regression results for overall social alienation.

Predictor	B	SE	β	T	*p*	95% CI for B	VIF
Digital Capital	−0.58	0.05	−0.61	−11.20	< 0.001	[−0.68, −0.48]	1.24
Sex	−0.14	0.06	−0.12	−2.32	0.021	[−0.26, −0.02]	1.08
Age	−0.05	0.04	−0.07	−1.34	0.180	[−0.13, 0.03]	1.11
Academic Level	−0.06	0.03	−0.09	−1.68	0.094	[−0.13, 0.01]	1.16

Model Summary: *N* = 320, R = 0.63, R^2^ = 0.397, Adjusted R^2^ = 0.390, F(4, 315) = 52.40, *p* < 0.001. B, unstandardized regression coefficient; SE, standard error; β, standardized regression coefficient; CI, 95% confidence interval for B; VIF, variance inflation factor.

Among the control variables, sex demonstrated a small but statistically significant negative association with social alienation (*β* = −0.12, *p* = 0.021), whereas age (*β* = −0.07, *p* = 0.180) and academic level (*β* = −0.09, *p* = 0.094) were not statistically significant. These findings indicate that the negative association between digital capital and social alienation remained statistically significant after accounting for key demographic and academic characteristics. Diagnostic checks further indicated no evidence of problematic multicollinearity among the predictors, with all variance inflation factor (VIF) values ranging from 1.08 to 1.24, well below commonly accepted thresholds, supporting the stability and adequacy of the estimated model.

## Discussion

The present study represents one of the relatively few empirical investigations examining the relationship between digital capital and social alienation within an Arab university context, particularly in the United Arab Emirates. In doing so, it contributes to the emerging literature by examining competence-based digital capital as a form of capital associated with students’ social integration and subjective experiences within contemporary higher education environments.

The findings indicated that undergraduate students at the participating university reported high levels of digital capital, reflecting a strong capacity to mobilize digital competencies across information and data literacy, communication and collaboration, digital content creation, safety, and problem solving. This finding is consistent with recent evidence from the United Arab Emirates demonstrating relatively high levels of digital competence among younger populations within a technologically advanced and highly connected environment (Muschert and Shomotova, 2025). At the same time, the UAE study showed that meaningful variations in digital competence continue to exist according to socioeconomic and demographic characteristics, suggesting that digital capital remains a socially embedded and differentiated resource even within digitally mature contexts. Given the competence-based operationalization adopted in the present study, these findings suggest that students possessed relatively strong digital competencies that constitute an important component of digital capital within the contemporary digital environment. However, these psychological constructs were not directly assessed in the present study.

This interpretation is consistent with previous studies indicating that university students generally possess relatively high levels of digital capital and digital competencies, providing them with opportunities to engage more effectively in academic and social activities (Haq and Mahmood, 2023; Lybeck et al., 2023).

From a theoretical standpoint, these findings can be interpreted through the framework of Pierre Bourdieu, who conceptualized capital as a set of accumulated resources that enable individuals to maintain or improve their position within social fields (Bourdieu, 1986). Within this perspective, digital capital can be understood as an emerging form of capital that extends beyond economic, cultural, and social capital to encompass digital competencies and digital technologies that can be mobilized to generate meaningful social, educational, and economic outcomes (Ragnedda, 2018; Ragnedda and Ruiu, 2020). In the context of higher education, digital capital may facilitate students’ ability to navigate academic environments, access institutional resources, and engage in meaningful academic and social interactions. Within a Bourdieusian perspective, these capacities may contribute to students’ social integration and lower experiences of social alienation. These theoretical interpretations are offered to contextualize the findings and should not be interpreted as empirically tested mechanisms in the present study.

In contrast, the findings indicated that the overall level of social alienation among students was low, suggesting generally satisfactory levels of social integration within the university environment. All dimensions of social alienation were reported at low levels, with meaninglessness emerging as relatively more prominent and social isolation as the least pronounced. This pattern may suggest that, although students were generally well integrated socially, perceptions of meaning and purpose remained relatively more salient than the other dimensions of social alienation.

The relatively low level of social alienation observed in this study may be interpreted in light of the contextual characteristics of the university environment. As a relatively small and recently established institution, the university may provide increased opportunities for direct interaction and the development of closer interpersonal relationships among students. In addition, the relative cultural and social homogeneity of the student population may contribute to a university environment characterized by shared norms and social cohesion, which may be associated with lower levels of social alienation. Although these contextual factors were not directly assessed in the present study, they are consistent with previous research emphasizing the importance of supportive academic and social environments in promoting student integration and reducing experiences of alienation (Chang et al., 2025; Gravett and Winstone, 2022).

Despite the overall low levels of social alienation, meaninglessness emerged as the most prominent dimension of alienation among students. This finding suggests that perceptions of meaning and purpose may remain relatively more salient than other dimensions of social alienation, even within a generally well-integrated university environment. Although previous research has shown that different patterns of digital engagement may be associated with psychological well-being (Verduyn et al., 2017; Valkenburg et al., 2022), the present study did not assess patterns of digital engagement. Therefore, these findings should be interpreted cautiously, and future research should examine the factors associated with meaninglessness among university students in digitally advanced higher education contexts.

One possible theoretical interpretation of the relatively higher level of meaninglessness is that, although students reported generally low levels of social alienation, perceptions of meaning and purpose may be shaped by factors beyond digital capital. Previous research suggests that experiences of meaning are influenced by multiple personal, educational, and social factors (Osin, 2015). Because these factors were not examined in the present study, this interpretation should be regarded as theoretical rather than empirical. Future research should investigate additional factors associated with meaninglessness among university students.

The negative association between digital capital and social alienation can also be interpreted through a Bourdieusian perspective. Digital capital may enhance students’ capacity to access information, communicate effectively, and participate in academic and social activities, thereby reducing barriers that may otherwise contribute to experiences of social alienation. Furthermore, digital capital may facilitate the development of social capital through digitally mediated interactions and online networks (Ellison et al., 2007), potentially supporting students’ social integration. This interpretation is consistent with recent evidence from the United Arab Emirates showing that digital inequalities remain embedded within broader patterns of social stratification despite advanced digital infrastructure and widespread connectivity (Muschert and Shomotova, 2025). Taken together, these findings suggest that digital capital may represent an important dimension of students’ capacity to participate effectively in academic and social life within digitally mediated environments.

This interpretation is also consistent with the digital divide framework proposed by Jan van Dijk, which suggests that digital inequalities extend beyond differences in access to encompass disparities in digital competencies, patterns of use, and the benefits derived from digital engagement (van Dijk, 2020; van Deursen and van Dijk, 2019). From this perspective, students with higher levels of digital capital may be better positioned to translate their digital competencies into more effective academic and social participation, whereas those with lower levels of digital capital may derive fewer benefits from digitally mediated environments.

Empirical support for this interpretation is provided by studies demonstrating that digital competencies are negatively associated with loneliness and social disconnection (Ko et al., 2022; Smith and Alheneidi, 2023) and that digital capital is positively associated with social support and psychological well-being (Alrawashdeh, 2025). Similarly, Clamor and Saloma (2023) reported that digital capital was positively associated with students’ sense of belonging, suggesting that digital capital may contribute to social integration and, consequently, lower experiences of social alienation.

With regard to demographic differences, the findings revealed that female students reported higher levels of digital capital than their male counterparts. This finding may reflect differences in the ways digital competencies are developed and applied across everyday academic and social contexts. Previous research has similarly suggested that digital inequalities are shaped not only by differences in access to technology but also by variations in digital competencies and patterns of digital use (Lybeck et al., 2023). In contrast, no statistically significant sex differences were found in social alienation, suggesting that male and female students reported comparable levels of social alienation within the university environment. This finding is consistent with previous research indicating that experiences of social alienation are influenced by multiple contextual and interpersonal factors beyond sex (Öksüz and Öztürk, 2017; Chang et al., 2025).

Furthermore, the absence of statistically significant differences across age groups may be understood in light of the relative homogeneity of the sample, which consisted primarily of young adults who shared broadly similar experiences with digital technologies. This finding is consistent with previous research suggesting that digital capital tends to be relatively homogeneous among younger populations who have grown up in digitally saturated environments (Alrawashdeh, 2025).

However, statistically significant differences were observed across academic levels, with higher levels of digital capital reported among students in more advanced years of study. This finding may reflect the cumulative nature of capital, as conceptualized by Pierre Bourdieu, whereby competencies and resources develop through continued experience and exposure. As students progress academically, they are likely to accumulate greater experience in applying digital competencies across academic contexts, which may contribute to higher levels of digital capital. This interpretation is consistent with previous studies emphasizing the importance of educational experiences in the development of digital competencies (Lybeck et al., 2023; Haq and Mahmood, 2023).

In contrast, no statistically significant differences were found in social alienation across academic levels, suggesting that students reported comparable levels of social alienation regardless of their year of study. This finding is consistent with previous research indicating that experiences of social alienation are shaped by multiple personal, social, and contextual factors rather than academic progression alone (Chen et al., 2025).

Overall, the findings suggest that digital capital may represent an important dimension of students’ capacity to participate effectively in academic and social life within digitally mediated university environments. At the same time, the findings indicate that the relationship between digital capital and social alienation is likely to be influenced by factors extending beyond digital capital alone. One possible interpretation is that broader social and institutional contexts may shape how digital capital is associated with experiences of social alienation. Because these factors were not directly assessed in the present study, these interpretations should be regarded as theoretical rather than empirical and warrant further investigation. Future research should therefore examine the mediating and moderating mechanisms, as well as the broader social and institutional contexts, that may help explain the relationship between digital capital and social alienation among university students.

### Theoretical and practical implications

The findings of this study carry important theoretical and practical implications for understanding the role of digital capital in contemporary higher education contexts. From a theoretical perspective, the results extend the application of digital capital theory within an Arab university setting by providing empirical support for its relevance as a multidimensional construct associated with students’ social integration and subjective experiences. By linking digital capital to the multidimensional conceptualization of social alienation proposed by Seeman (1959), the study offers a sociological perspective on how digital competencies may be associated with students’ experiences of social alienation within university environments. The findings further support the relevance of Pierre Bourdieu’s framework (Bourdieu, 1986), suggesting that digital capital operates as an extension of traditional forms of capital that facilitates participation within social fields. They also align with Jan van Dijk’s perspective that digital inequalities are shaped not only by access to technology but also by differences in digital competencies, patterns of use, and the ability to derive meaningful outcomes from digital engagement (van Dijk, 2020; van Deursen and van Dijk, 2019).

From a practical standpoint, the findings highlight the need for higher education institutions to move beyond providing technological infrastructure toward actively fostering advanced digital competencies among students. This may include integrating digital competencies into academic curricula, promoting interactive and collaborative digital learning environments, and encouraging meaningful and reflective forms of digital engagement. The relatively higher level of meaninglessness observed in the present study further suggests that digital competencies alone may be insufficient to promote meaningful integration. Universities may therefore benefit from adopting pedagogical approaches that emphasize purpose-driven learning by connecting academic experiences with students’ personal goals and future aspirations while strengthening academic advising and psychological support services. In addition, previous research has suggested that digital social support may be associated with lower levels of alienation (Alrawashdeh, 2025). Accordingly, universities may benefit from leveraging digital platforms to foster supportive online communities that encourage interaction, peer engagement, and emotional connectedness. Institutions may also consider integrating digitally supported collaborative learning activities that promote collaboration, knowledge sharing, and students’ sense of belonging within online academic communities. Given that students in the lower academic levels reported significantly lower digital capital than their senior peers, universities may particularly benefit from introducing structured digital competency initiatives during the first years of study. Such initiatives may facilitate students’ academic adaptation and strengthen their engagement with digitally mediated learning environments during the transition into university.

Taken together, these implications suggest that digital capital represents more than the acquisition of digital competencies and may constitute an important resource for promoting students’ academic and social integration within digitally mediated university environments.

### Limitations

Despite the contributions of this study, several methodological limitations should be acknowledged. First, the cross-sectional design precludes causal inferences regarding the relationship between digital capital and social alienation, as the findings reflect associations observed at a single point in time rather than longitudinal processes. Second, the study relied on self-reported data, which may be subject to response biases, including social desirability and recall bias, potentially affecting the accuracy of participants’ responses. Moreover, because all variables were measured using self-report questionnaires administered to the same participants, the possibility of common method bias cannot be entirely ruled out. Although procedural and statistical measures were undertaken to reduce and assess this risk, the observed associations should be interpreted with appropriate caution.

Third, the study employed convenience sampling and was conducted at a single government-affiliated university in the Emirate of Sharjah, United Arab Emirates. The student population was characterized by relative cultural and social homogeneity, which may limit the generalizability of the findings beyond this institutional context. Accordingly, the findings should not be interpreted as representative of all undergraduate students in the United Arab Emirates.

In addition, although the study employed previously established measures that demonstrated satisfactory reliability and underwent careful translation, cultural adaptation, expert review, and pilot testing procedures, reliance on quantitative instruments alone may not fully capture the complexity of students’ lived experiences of digital engagement and social alienation. Furthermore, although digital capital is theoretically conceptualized as encompassing both digital access and digital competencies, the present study operationalized digital capital primarily through students’ digital competencies. Consequently, the findings should be interpreted within the scope of this operationalization and may not fully reflect other theoretical dimensions of digital capital. Although the psychometric evidence obtained in the present study supported the reliability and internal consistency of the measures, future research may further examine their construct validity using additional psychometric approaches, such as confirmatory factor analysis and other measurement validation procedures across diverse educational and cultural contexts.

Finally, although the analyses controlled for key demographic variables (sex, age, and academic level) based on previous literature, other theoretically relevant factors, such as socioeconomic status, academic performance, perceived social support, personality characteristics, academic motivation, family environment, and broader institutional conditions, were not included in the analysis and may therefore limit the comprehensiveness of the explanatory model. Future research should examine the contribution of these factors to provide a more comprehensive understanding of the relationship between digital capital and social alienation among university students.

## Conclusion

This study examined the relationship between digital capital and social alienation among undergraduate students at a university in the United Arab Emirates. The findings indicated that higher levels of digital capital were associated with lower levels of perceived social alienation across all dimensions, with the strongest association observed for normlessness, followed by social isolation, meaninglessness, and powerlessness. At the same time, the relatively higher level of meaninglessness suggests that digital capital alone may be insufficient to address all dimensions of students’ experiences of social alienation.

Overall, the findings support the relevance of digital capital as a multidimensional resource associated with students’ social integration and experiences of social alienation within digitally mediated higher education environments. They further suggest that the potential benefits of digital capital extend beyond the acquisition of digital competencies and may contribute to broader processes of academic and social integration. Future research should employ longitudinal and mixed-method designs to examine the mechanisms through which digital capital may influence different dimensions of social alienation and to explore the contribution of additional contextual and psychosocial factors across diverse educational and cultural settings.

## Data Availability

The original contributions presented in the study are included in the article/supplementary material, further inquiries can be directed to the corresponding author.
